# The *Escherichia coli* Cryptic Prophage Protein YfdR Binds to DnaA and Initiation of Chromosomal Replication Is Inhibited by Overexpression of the Gene Cluster *yfdQ*-*yfdR*-*yfdS*-*yfdT*

**DOI:** 10.3389/fmicb.2016.00239

**Published:** 2016-03-03

**Authors:** Yasunori Noguchi, Tsutomu Katayama

**Affiliations:** Department of Molecular Biology, Graduate School of Pharmaceutical Sciences, Kyushu UniversityFukuoka, Japan

**Keywords:** DnaA, replication initiation, cryptic prophage, *E. coli*, regulation of initiation

## Abstract

The initiation of bacterial chromosomal replication is regulated by multiple pathways. To explore novel regulators, we isolated multicopy suppressors for the cold-sensitive *hda-185* Δ*sfiA(sulA)* mutant. Hda is crucial for the negative regulation of the initiator DnaA and the *hda-185* mutation causes severe replication overinitiation at the replication origin *oriC*. The SOS-associated division inhibitor SfiA inhibits FtsZ ring formation, an essential step for cell division regulation during the SOS response, and Δ*sfiA* enhances the cold sensitivity of *hda-185* cells in colony formation. One of the suppressors comprised the *yfdQ*-*yfdR*-*yfdS*-*yfdT* gene cluster carried on a cryptic prophage. Increased copy numbers of *yfdQRT* or *yfdQRS* inhibited not only *hda-185-*dependent overinitiation, but also replication overinitiation in a hyperactive *dnaA* mutant, and in a mutant lacking an *oriC*-binding initiation-inhibitor SeqA. In addition, increasing the copy number of the gene set inhibited the growth of cells bearing specific, initiation-impairing *dnaA* mutations. In wild-type cells, multicopy supply of *yfdQRT* or *yfdQRS* also inhibited replication initiation and increased hydroxyurea (HU)-resistance, as seen in cells lacking DiaA, a stimulator of DnaA assembly on *oriC*. Deletion of the *yfdQ*-*yfdR*-*yfdS*-*yfdT* genes did not affect either HU resistance or initiation regulation. Furthermore, we found that DnaA bound specifically to YfdR in soluble protein extracts oversupplied with YfdQRST. Purified YfdR also bound to DnaA, and DnaA Phe46, an amino acid residue crucial for DnaA interactions with DiaA and DnaB replicative helicase was important for this interaction. Consistently, YfdR moderately inhibited DiaA-DnaA and DnaB-DnaA interactions. In addition, protein extracts oversupplied with YfdQRST inhibited replication initiation *in vitro*. Given the roles of *yfdQ* and *yfdS* in cell tolerance to specific environmental stresses, the *yfdQ*-*yfdR*-*yfdS*-*yfdT* genes might downregulate the initiator DnaA-*oriC* complex under specific growth conditions.

## Introduction

Chromosomal replication initiation is tightly regulated to allow cell cycle progression, and its regulation is one of the important targets of stress responses (Sclafani and Holzen, 2007; O’Donnell et al., 2013). Activities of several proteins responsible for the initiation are controlled during the cell cycle (Katayama et al., 2010; Leonard and Grimwade, 2015; Marczynski et al., 2015; Wolanski et al., 2015). However, our knowledge of the initiation regulation mechanisms is still limited, and the pathways, which regulate the initiation under various cellular circumstances, remain to be explored.

In *Escherichia coli*, the initiator protein DnaA recognizes the chromosomal replication origin, *oriC* (Ozaki and Katayama, 2009; Kaguni, 2011; Leonard and Grimwade, 2011; Costa et al., 2013; Saxena et al., 2013). DnaA has a high affinity for both ATP and ADP (Sekimizu et al., 1987; Saxena et al., 2015). ATP-bound DnaA (ATP-DnaA), but not ADP-bound DnaA (ADP-DnaA), forms stable multimers on *oriC* and induces unwinding of a specific AT-rich *oriC* region, depending on the *oriC* binding of the nucleotide-associating protein IHF (Ryan et al., 2002; Dillon and Dorman, 2010; Ozaki and Katayama, 2012). A DnaA-binding protein DiaA stimulates DnaA assembly on *oriC* and *oriC* unwinding (Ishida et al., 2004; Keyamura et al., 2007, 2009). Deletion of the *diaA* gene causes inhibition of the replication initiation; i.e., when multiple sister *oriC* copies are present in rapidly growing *diaA* mutant cells, initiation at each *oriC* occurs asynchronously due to delays in initiation at each *oriC*. After *oriC* unwinding, DnaB helicase is loaded onto the resultant single-stranded DNA (ssDNA) via interactions with the *oriC*-bound DnaA multimers and DnaC helicase loader (Kaguni, 2011). DiaA is proposed to be dissociated from DnaA-*oriC* complexes when DnaB interacts with DnaA (Keyamura et al., 2009). The loaded DnaB helicases recruit DnaG primases and DNA polymerase III holoenzymes onto DNA, forming replisomes for the synthesis of nascent DNA strands. After Okazaki fragment synthesis on the lagging strand, the clamp subunit dissociates from DNA polymerase III and remains temporarily loaded on the lagging strand (Langston et al., 2009; Su’etsugu and Errington, 2011; Moolman et al., 2014).

The cellular levels of ATP-DnaA oscillate during the cell cycle (Kurokawa et al., 1999). The ATP-DnaA levels peak before replication initiation, and decrease after the initiation. The decrease in ATP-DnaA levels is predominantly dependent on the RIDA (regulatory inactivation of DnaA) system, in which the DnaA-bound ATP is hydrolyzed by Hda protein complexed with the DNA-loaded clamp, resulting in the inactive ADP-DnaA (Katayama et al., 2010). Hda comprises a short N-terminal region containing a clamp-binding motif and an AAA+ family domain, which includes a specific motif stimulating ATP-DnaA hydrolysis (Kato and Katayama, 2001; Su’etsugu et al., 2008; Katayama et al., 2010; Nakamura and Katayama, 2010). Loss of Hda function *in vivo* causes the increase of the ATP-DnaA levels and replication overinitiation, resulting in inhibition of cell growth and replication fork instability (Kato and Katayama, 2001; Fujimitsu et al., 2008; Charbon et al., 2014).

Recently, *datA* region of the chromosome was reported to assist RIDA in decreasing the ATP-DnaA levels (Kasho and Katayama, 2013). This region contains a DnaA box cluster and an IHF-binding site. DnaA-bound ATP hydrolysis on this region occurs in a timely manner during the cell cycle and depends on the temporal binding of IHF to *datA*.

In addition to the pathways that stimulate DnaA-bound ATP hydrolysis, the interaction of DnaA with *oriC* is inhibited immediately after replication initiation (Waldminghaus and Skarstad, 2009). Although *E. coli* chromosomal DNA is methylated for most of the cell cycle, there exists a brief period after the synthesis of nascent strands when the DNA is hemimethylated. SeqA preferentially binds to hemimethylated *oriC*, which prevents DnaA from re-binding to *oriC*, and inhibits inappropriate re-initiations (Nievera et al., 2006; Fossum-Raunehaug et al., 2014). The *seqA* null mutant cells exhibit a moderate replication overinitiation, although they grow at a rate similar to that of wild-type cells (Lu et al., 1994).

ATP-DnaA is generated in the course of *de novo* DnaA synthesis or ADP-DnaA reactivation (Fujimitsu et al., 2009; Kasho et al., 2014). Most of the *de novo* synthesized DnaA should bind ATP, which is more abundant in cells than ADP. ATP-DnaA is also re-generated at the chromosomal regions *DARS1* (DnaA-reactivating sequence 1) and *DARS2*, via nucleotide exchange from ADP-DnaA, the product of the DnaA-bound ATP hydrolysis mentioned above (Fujimitsu et al., 2009). Both *DARS1* and *DARS2* contain a mutually similar DnaA box cluster, and *DARS2* is activated by IHF binding (Fujimitsu et al., 2009; Kasho et al., 2014). Acidic phospholipids may also contribute to the re-generation of ATP-DnaA from ADP-DnaA (Fingland et al., 2012).

DnaA consists of four functional domains (Ozaki and Katayama, 2009; Kaguni, 2011). Domain I interacts with several proteins and forms a homodimer (Felczak et al., 2005; Abe et al., 2007; Keyamura et al., 2009). Phe46 residue of this domain is responsible for binding DiaA and DnaB (Abe et al., 2007; Keyamura et al., 2009). Domain II is a flexible linker (Abe et al., 2007; Nozaki and Ogawa, 2008). Domain III contains a set of motifs specific for the AAA+ family and plays various roles in ATP/ADP binding, ATP hydrolysis, ssDNA binding, and DnaA-multimer formation (Erzberger et al., 2002; Nishida et al., 2002; Felczak and Kaguni, 2004; Kawakami et al., 2005, 2006; Ozaki et al., 2008, 2012). Domain IV binds DNA in a sequence-specific manner (Roth and Messer, 1995; Erzberger et al., 2002; Fujikawa et al., 2003).

We previously isolated an *E. coli hda-185* mutant, a cold-sensitive mutant of *hda* (Fujimitsu et al., 2008). The *hda-185* mutant carries a mutation in the AAA+ motif and is characterized by elevated cellular ATP-DnaA levels at 25°C. The *hda-185* cells thus exhibit overinitiation of chromosomal replication from *oriC* at 25°C, which is accompanied by impeded progression of the replication forks and the inhibition of colony formation. The *hda-185*-specific inhibition of colony formation is further enhanced by a disruption of *sfiA (sulA)* gene, which encodes an SOS protein that inhibits FtsZ activity in cell division (Mukherjee et al., 1998; Fujimitsu et al., 2008).

In this study, we isolated a set of genes, *yfdQ*-*yfdR*-*yfdS*-*yfdT*, as a multicopy suppressor of the *hda-185* Δ*sfiA* cells. This gene set is carried by a cryptic prophage CPS-53 located in the terminus domain of the chromosome (Wang et al., 2010). *yfdQ* and *yfdS* enhance bacterial resistance to the alkylating agent methyl methanesulfonate (MMS) and oxidative stress, respectively (Rooney et al., 2009; Wang et al., 2010). None of the four genes have been characterized in the context of chromosomal replication. Here, we report that increasing the copy number of these genes inhibited *hda-185-*dependent overinitiation, in addition to replication initiation in wild-type cells. Also, the increased copy number inhibited the growth of temperature-sensitive *dnaA* mutants at 30°C in an allele-specific manner. Deletion of the gene set did not significantly affect replication regulation in cells growing at 37°C. In a pull-down assay, DnaA specifically bound to YfdR in a soluble protein extract oversupplied with YfdQRST. The purified YfdR protein bound DnaA directly and DnaA-domain I Phe46 is suggested to play an important role in this interaction. In addition, a set of the suppressor proteins inhibited replication initiation at *oriC in vitro*. Based on these results, we discuss the potential roles for *yfdQ*-*yfdR*-*yfdS*-*yfdT* genes in the regulation of replication initiation.

## Materials and Methods

### Strains

*Escherichia coli* strains are listed in **Table 1**. MIT125, NY10, NY11, NY12, MIT140, MIT143, MIT162, and MIT147 are derivatives of MG1655 constructed by P1 transduction using phage lysates of KA452, NKN211, NKN212, NKN241, NKN1, NKN243, YT411, and KA483, respectively (Su’etsugu et al., 2003; Ishida et al., 2004; Fujimitsu et al., 2008). The *dnaA*5 *tnaA*::*Tn*10 or *dnaA*204 *tnaA*::*Tn*10 mutations were transferred via P1 transduction into MIT162, resulting in NY16 or NY17, respectively. JW3118 was obtained from the Keio collection and used for P1 transduction-construction of SA103. For NY5, *frt*-*kan* fragment was PCR-amplified using pTH5 as template, and primers nogu100 (5′-GAATATCTTAATATAGTGAGGACTTATTATGTCTCAGAACTTAGACGCAACCGCAAGTGTAGGCTGGAGCTGCTTC-3′) and nogu101 (5′-GATGATCCAACCGAGAGTCATATCCCATGCCATGTATTCGTTATCGCCGTTTTTTGCATATGAATATCCTCCTTAG-3′). This fragment was introduced into MG1655 using the λRed recombination system, as previously described (Datsenko and Wanner, 2000; Noguchi et al., 2015). MIT123 was constructed using MIT84 and *cat* gene from pACYC184 by a method similar to that used for constructing MIT92, as described elsewhere (Fujimitsu et al., 2009).

**Table 1:** Strain list.

Name	Genotype	Reference
MG1655	Wild-type	Laboratory stock
MZ001	MG1655 *thyA rpsL hda-185 kan* Δ*sfiA*	Fujimitsu et al., 2008
YH013	MG1655 *thyA rpsL hda-185 kan*	Fujimitsu et al., 2008
YH014	MG1655 *thyA rpsL hda*^+^ *kan*	Fujimitsu et al., 2008
MC061	MG1655 *araD139* Δ(*araABC-leu*)*7679*	Ishida et al., 2004
MIT125	MG1655 *dnaA46 tnaA*::Tn*10*	This work
NY10	MG1655 *dnaA5 tnaA*::Tn*10*	This work
NY11	MG1655 *dnaA167 tnaA*::Tn*10*	This work
NY12	MG1655 *dnaA601 tnaA*::Tn*10*	This work
MIT140	MG1655 *dnaA508 tnaA*::Tn*10*	This work
MIT143	MG1655 *dnaA204 tnaA*::Tn*10*	This work
MIT162	MG1655 *rnhA*::*cat*	This work
NY16	MG1655 *dnaA5 tnaA*::Tn*10 rnhA*::*cat*	This work
NY17	MG1655 *dnaA204 tnaA*::Tn*10 rnhA*::*cat*	This work
KH5402-1	*ilv thyA tyrA*(Am) *trpE9829*(Am) *metE deo supF6*(Ts)	Fujimitsu et al., 2008
NA001	KH5402-1 *dnaAcos*	Fujimitsu et al., 2008
NY5	MG1655 Δ*yfdQ-yfdR-yfdS-yfdT* ::*kan*	This work
MIT147	MG1655 *seqA*::Tn*10*	This work
SA103	MG1655 Δ*diaA*::*kan*	This work
JW3118	BW25113 Δ*diaA*::*kan*	NIG
MIT123	MG1655 Δ*DARS1*::*cat* Δ*DARS2*::*spec*	This work
NT26	MG1655 Δ*dnaA*::*spec rnhA*::*kan*	Noguchi et al., 2015
ME5491	*dnaB43*(Ts)	NIG


### Plasmids

Plasmids pNA135 and pBRoriC were previously described (Ishida et al., 2004; Noguchi et al., 2015). Plasmid pSU17 was isolated as a suppressor of *hda-185* cells. To construct pSU17 derivatives, pSU17 was digested with restriction enzymes *Stu*I, *Eco*RI, *Sph*I, or *Bst*BI, and then self-ligated, resulting in pST17, pEC17, pSP17, or pBS17, respectively. To construct plasmid pSS17, pSU17 was digested with *Stu*I and *Sph*I, blunted using Blunting high kit (TOYOBO), and self-ligated. To construct pSS17 derivatives, pSS17 was digested with *Pac*I and *Bam*HI, or *Hpa*I and *Bam*HI, accordingly, blunted, and self-ligated, resulting in pSSP or pSSPH, respectively. To construct pSSP derivatives, pSSP was digested with *Nsi*I or *Bst*BI, blunted, and self-ligated, resulting in pSSPN or pSSPB, respectively. To construct plasmid pSSPT, pSSP template was PCR-amplified with primers nogu5 (5′-TTGAGGATCCGGCTGCTGATTCGTTCTTTG-3′) and nogu6 (5′-AAGTTGGATCCTATCGACTACGCGATCATGG-3′), and the resulting product was digested with *Bam*HI, and ligated with pBR322 vector digested with the same restriction enzyme. To construct pSPTB, pSSPT was digested with *Bst*BI, blunted, and self-ligated.

To construct plasmid pQRST carrying the *yfdQ*, *yfdR*, *yfdS*, and *yfdT* genes, pSSP template was PCR-amplified with primers nogu7 (5′-GCGCTGCTAGCGTGAGGACTTATTATGTCTC-3′) and nogu9 (5′-CATATAAGCTTCGAGTGTGAGGCTGTATGGC-3′), digested with *Hin*dII and *Nhe*I, and ligated with pBAD18 vector digested with the same restriction enzymes.

To construct plasmid pYfdR for YfdR protein overproduction, pSSP template was PCR-amplified with primers nogu10 (5′-AATTAGCTAGCATGTCATTTATTAAAAC-3′) and nogu11 (5′-CTGATGAATTCCGAAAACATTCATG-3′), digested with *Eco*RI and *Nhe*I, and ligated with pBAD/HisB vector (Invitrogen) digested with the same restriction enzymes.

### Buffers

Buffer NY2 contained 50 mM Hepes-KOH (pH 7.6), 2 mM DTT, 0.1 mM EDTA, and 50 mM NaCl. Buffer C contained 50 mM Hepes-KOH (pH 7.6), 1 mM EDTA, 2 mM DTT, 20% sucrose, 100 mM KCl, 10 mM magnesium acetate, 0.01% Brij-58, 0.1 mg/mL bovine serum albumin, and 10 μM ATP. Buffer NY6 contained 50 mM Hepes-KOH (pH 7.6), 0.1 mM EDTA, 10% glycerol, 100 mM NaCl, and 2 mM β-mercaptoethanol. Buffer R contained 20 mM Tris-HCl (pH 7.5), 1 mM EDTA, 4 mM β-mercaptoethanol, 10% glycerol, 50 mM KCl, 5 mM magnesium acetate, 0.1% Triton-X100, 0.1 mg/mL bovine serum albumin, and 2 mM ATP. Buffer M contained 20 mM Tris-HCl (pH 7.5), 0.1 mg/mL bovine serum albumin, 8 mM DTT, 10 mM magnesium acetate, 125 mM potassium glutamate, and 2 mM ATP.

### Preparation of Protein Extracts

MG1655 cells carrying pBAD18 or pQRST were grown at 37°C in LB medium. When the absorbance (A_660_) reached 0.5, arabinose (final concentration 0.5%) was added and the cells were incubated for a further 2 h, following which they were harvested by centrifugation at 4°C, incubated on ice for 30 min in buffer NY2 supplemented with 0.3 mg/mL lysozyme, and frozen in liquid nitrogen. The frozen cells were thawed on ice as required, and the supernatants were collected after centrifugation.

### Pull-Down Assay Using Biotin-Tagged DnaA (bio-DnaA)

This assay was performed as previously described (Ishida et al., 2004). Purification of bio-DnaA, bio-DnaA domain I–II, and bio-DnaA domain III–IV has also been reported (Ishida et al., 2004). Aliquots of protein extracts described above were incubated for 5 min on ice in buffer C (20 μL), in the presence or absence of bio-DnaA, or its truncated derivatives (10 pmol). The mixtures were further incubated for 1 h at 4°C, with gentle rotation, in the presence of streptavidin-conjugated magnetic beads (Promega). The beads and the bound materials were collected using magnetic force and washed twice with buffer C (20 μL) without bovine serum albumin. DnaA-bound proteins were eluted in a standard SDS sample buffer (10 μL) and analyzed using SDS-13% PAGE and silver staining.

### Purification of His-YfdR

MC1061 cells bearing pYfdR were grown at 37°C in LB medium containing 50 μg/mL ampicillin. When the absorbance (A_660_) of the culture reached 0.5, arabinose (final concentration 0.5%) was added, and the cultures were incubated for a further 2 h, harvested by centrifugation, incubated on ice for 30 min in buffer NY6 containing 10 mM imidazole and 0.3 mg/mL lysozyme, and frozen in liquid nitrogen. The frozen cells were thawed on ice, as required, and the supernatants were collected after centrifugation, and applied onto Ni-NTA agarose column (1 mL bed volume, Qiagen). The column was washed with NY6 buffer supplemented with 45 mM imidazole, and His-YfdR was eluted with buffer NY6 with 300 mM imidazole. Protein concentrations were determined using Bradford assay kit (Bio-Rad).

### Pull-Down Assays with His-YfdR

His-YfdR (10 pmol) was incubated for 5 min on ice in buffer R containing 20 mM imidazole (20 μL), in the presence or absence of DnaA, followed by further incubation for 15 min on ice with Co^2+^-conjugate magnetic beads (Dynabeads, Invitrogen). The beads and the bound materials were collected using magnetic force and washed with buffer R (20 μL) without bovine serum albumin but containing 100 mM KCl and 50 mM imidazole, accordingly. Proteins were eluted in standard SDS sample buffer (10 μL) and analyzed by SDS-13% PAGE and silver staining.

### Pull-Down Assays with a Biotin-Tagged *oriC* Fragment (bio-*oriC*)

This assay was performed as described previously (Keyamura et al., 2007, 2009; Ozaki and Katayama, 2012; Ozaki et al., 2012). Briefly, DnaA and bio-*oriC* were mixed and incubated with His-YfdR or protein extracts on ice for 10 min in buffer (10 μL), followed by further incubation at 4°C for 15 min in the presence of streptavidin-coated beads (Promega). The beads and bound materials were collected, and washed. The bound proteins were dissolved in SDS sample buffer, and analyzed by SDS-12% PAGE and silver staining. In the DiaA-YfdR competition assay, DnaA and bio-*oriC* were mixed and incubated with His-DiaA on ice for 5 min, followed by further incubation with His-YfdR for 10 min. The bound proteins were analyzed by SDS-15% PAGE. In DnaB-YfdR competition assay, DnaA and bio-*oriC* were mixed and incubated with DnaB-DnaC complex on ice for 5 min, followed by further incubation with His-YfdR for 10 min. The bound proteins were analyzed by SDS-12% PAGE.

### Form I^∗^ Assays

This analysis was performed basically as previously described (Noguchi et al., 2015). Briefly, protein extracts or purified His-YfdR were incubated on ice in buffer M (25 μL) containing SSB, IHF, DnaB, DnaC, gyrase, and supercoiled (form I) pBRoriC. The mixture was further incubated with ATP-DnaA, at 30°C for 15 min. Reactions were stopped by the addition of phenol and chloroform. DNA was precipitated with ethanol and dissolved in Tris-EDTA buffer, followed by 0.65% agarose gel electrophoresis and ethidium bromide staining. The relative amounts of form I^∗^ DNA were quantified by densitometry.

## Results

### Isolation of Multicopy *hda-185* Mutant Suppressors

We previously isolated the cold-sensitive mutant of *hda*, *hda-185* (Fujimitsu et al., 2008). MZ001 (*hda-185* Δ*sfiA*) cells induce overinitiation of chromosomal replication, and cell division is inhibited at 25°C, resulting in the inhibition of colony formation. Here, to explore novel regulatory pathways of replication initiation, we isolated multicopy suppressors of *hda-185* using pBR322-based DNA library containing chromosomal *Bam*HI fragments. MZ001 (*hda-185* Δ*sfiA*) cells were transformed with this DNA library, and incubated at 25°C on LB agar plates containing ampicillin. Nineteen independent transformants were confirmed as resistant to ampicillin and able to grow at 25°C.

We identified chromosomal regions carried by the plasmids by DNA sequencing. The regions were classified into three groups. Group 1 plasmids contained the *oriC* region and were recovered from 15 independent transformants. The *oriC* region was previously isolated as a suppressor of a *dnaA*cos mutant, which induced overinitiation of chromosomal replication and inhibited colony formation at 30°C (Katayama and Kornberg, 1994). DnaAcos protein is resistant to RIDA and sustains the replication initiation activity at 30°C, which leads to overinitiation of replication (Katayama, 1994; Katayama and Kornberg, 1994; Chodavarapu et al., 2013). It is suggested that the plasmid-borne multiple *oriC* copies titrate DnaA molecules, resulting in a decreased amount of DnaA available for chromosomal *oriC* binding and, therefore, inhibition of additional chromosomal replication initiations. Group 2 plasmids contained the *diaA* gene and were recovered from three independent transformants. DiaA protein functions as a positive and negative regulator of replication initiation. Both depletion and excess of DiaA restrict the replication initiation activity (Ishida et al., 2004; Keyamura et al., 2007). DiaA binds to DnaA directly, stimulating DnaA oligomerization at *oriC*. DiaA and DnaB bind to DnaA at the same site (i.e., the site including Phe46), and therefore DiaA competes for DnaA binding with the other protein (Keyamura et al., 2009). Thus, it is thought that excess amounts of DiaA inhibit replication initiation by preventing DnaB loading (Ishida et al., 2004; Keyamura et al., 2009).

**FIGURE 1:**
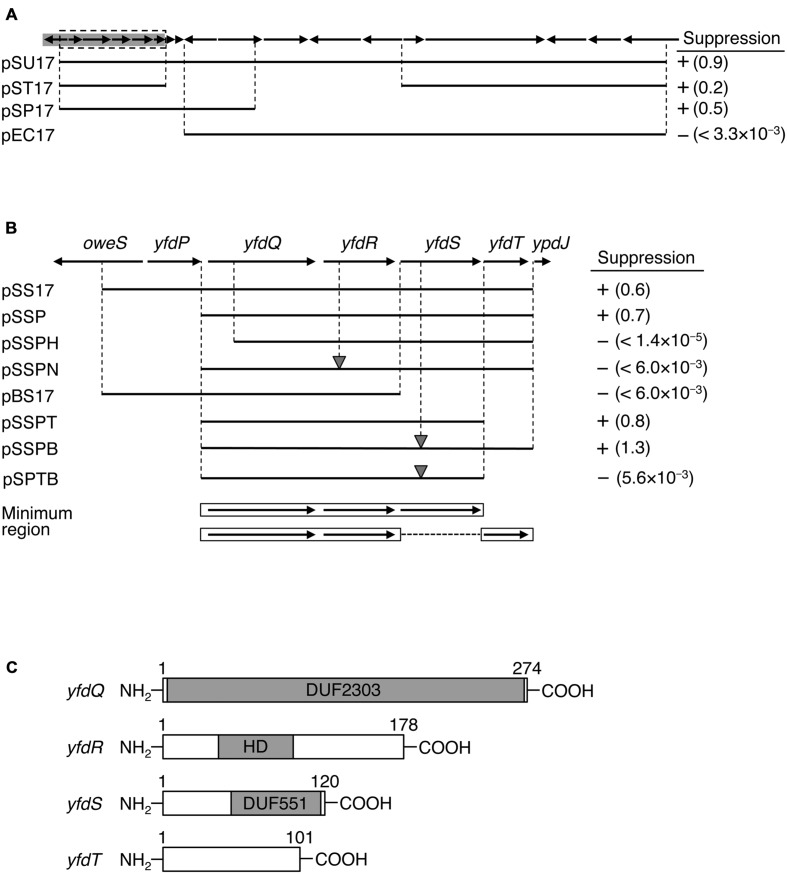
**Identification of suppressor genes.** **(A)** Map of ORFs from 53.26 to 53.63 min of the *Escherichia coli* genome. ORFs are shown as arrows indicating the direction of transcription. Prophage CPS-53 element fragment is highlighted in gray. Genomic regions carried on each plasmid are shown as black bars. Colony-forming ability of MZ001 (*hda-185* Δ*sfiA*) cells carrying the respective plasmids at 25°C or 42°C was analyzed, and the results are indicated as “+” (suppressed) or “-” (not suppressed). Numbers in parentheses indicate the ratio of transformation efficiencies at 25°C and 42°C. **(B)** Enlarged gray region from **(A)**. DNA fragments carried by plasmids and colony formation are indicated as above. The gray triangles indicate frame shift mutations. Minimal regions required for suppression are indicated at the bottom with open boxes and arrows. **(C)** Protein motifs for YfdQRST were deduced using Pfam database and are indicated by gray boxes. DUF2303 and DUF551 are domains of unknown function and are conserved in many proteins deposited in the database. HD refers to HD domain. Amino acid numbers of each protein product are also given.

Group 3 plasmid included a fragment derived from the 53.26–53.63 min genetic map region (pSU17 of **Figure 1A**) and was recovered from a single independent transformant. The region contains 16 ORFs (Yamazaki et al., 2008) and none of these were previously reported as regulating replication initiation. We therefore focused our investigation on pSU17 plasmid from Group 3 (**Figure 1A**).

### Identification of Genes Responsible for *hda-185* Suppression

To determine the DNA fragment responsible for *hda-185* suppression, we constructed plasmid derivatives of pSU17 (**Figures 1A,B**). An initial deletion analysis indicated that derivatives containing either the five 5′ ORFs and three 3′ ORFs (pST17), or eight 5′ ORFs (pSP17), all exerted the suppression effect, whereas a derivative lacking the seven 5′ ORFs (pEC17) did not (**Figure 1A**). These results suggested that the five 5′ ORFs, but not the eleven 3′ ORFs, were responsible for suppression. This was confirmed by the observation that plasmid pSS17 carrying only the five 5′ ORFs, *yfdP-yfdT*, sustained the suppression ability (**Figure 1B**). Further analysis of pSSP, a deletion derivative of pSS17, showed that *yfdP* was not required for suppression.

Analysis of pSSPH showed that *yfdQ* was required for suppression of *hda-185* (**Figure 1B**). Furthermore, the *yfdR* frame shift mutation in the pSSP-derived pSSPN revealed that this gene was also required for the suppression. Plasmid pBS17, which carries *yfdQR* but not *yfdST*, did not suppress *hda-185*. Further analysis was performed using pSSP derivatives, pSSPT and pSSPB, lacking *yfdT* or possessing a frame shift mutation in *yfdS*, respectively. These two plasmids suppressed *hda-185*, but plasmid pSPTB, a pSSPB derivative lacking *yfdT*, did not (**Figure 1B**). These results indicated that either *yfdT* or *yfdS* were required for suppression of *hda-185*. Taken together, the minimum gene set required for *hda-185* suppression comprised *yfdQ*, *yfdR*, and either *yfdS* or *yfdT*. In addition, the results with the frame shift mutants suggest that the suppression is due to the protein products YfdR and YfdS, but not the *yfdR* and *yfdS* DNA sequences themselves.

Previous genome analysis indicated that all the investigated genes, i.e., *yfdQ*, *yfdR*, *yfdS*, and *yfdT*, reside on a cryptic prophage called CPS-53, but the functions of these phage-derived genes are yet to be experimentally explored (Yamazaki et al., 2008). Pfam motif database analysis indicated that YfdQ is mostly comprised of a domain of unknown function (**Figure 1C**, DUF2302) (Finn et al., 2014), although *yfdQ*-deletion mutants were weakly sensitive to MMS and H_2_O_2_ (Rooney et al., 2009; Wang et al., 2010). YfdR contains an HD domain that includes specific His and Asp residues and is common to various nucleotide phosphohydrolases (Aravind and Koonin, 1998). In *E. coli*, an HD domain protein YfbR has been characterized as a functional phosphohydrolase for nucleoside mono-phosphates required in *de novo* thymidylate synthesis pathway (Weiss, 2007; Zimmerman et al., 2008). Although YfdR is a structural homolog of YfbR, the nucleotide affinities of YfdR are much lower than those of YfbR, and thus the function of YfdR remains elusive (Zimmerman et al., 2008). YfdS contains an uncharacterized motif (DUF551) that is also found in various proteins specific for double-strand DNA viruses with no RNA-dependent development stage, such as λ and P22 phages, and for some *E. coli* cryptic prophages (Finn et al., 2014). Cells lacking *yfdS* are sensitive to H_2_O_2_ (Wang et al., 2010). YfdT does not have a defined protein motif.

### *yfdQRST* Inhibits the Chromosomal Replication Initiation

**FIGURE 2:**
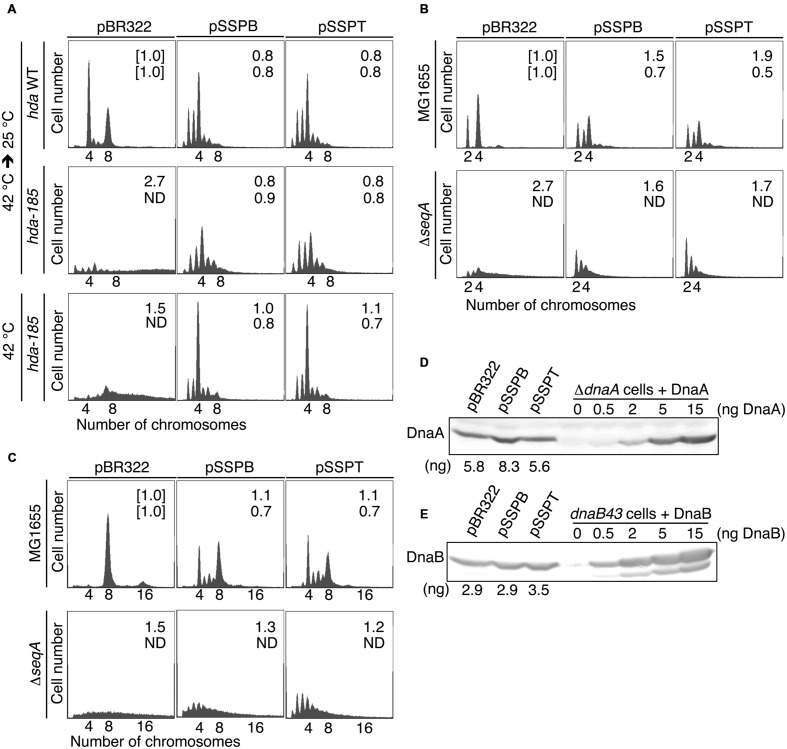
**pBR322-*yfdQRST* inhibits the initiation of chromosomal replication.** **(A)** YH014 (*hda* WT) and YH013 (*hda-185*) cells carrying pBR322, pSSPB, or pSSPT were exponentially grown in LB medium containing 50 mg/mL thymine at 42°C, and further incubated at 25°C for five generations. Some culture aliquots were used to determine cell size (cell mass) by flow cytometry. The remaining aliquots were further incubated for 4 h in the presence of rifampicin and cephalexin, followed by DNA content quantification with flow cytometry. Chromosome numbers corresponding to the detected peaks are shown. Mean cell mass (*upper*) and the *oriC* number per cell mass (*lower*) relative to YH014 wild-type cells bearing pBR322 is indicated at the top right corner of each panel. N.D., not determined because of non-discrete peaks of DNA content. **(B,C)** MG1655 and MIT147 (Δ*seqA*) cells carrying pBR322, pSSPB, or pSSPT were grown in M9 medium containing 0.2% glucose and 0.2% casamino acids **(B)** or LB medium **(C)**, at 37°C, and analyzed using flow cytometry. Mean cell mass (*upper*) and the *oriC* number per cell mass (*lower*) relative to that of MG1655 cells bearing pBR322 is indicated at the top right corner of each panel. **(D,E)** MG1655 cells carrying the indicated plasmid were grown in LB medium at 37°C to an absorbance (A_660_) of 0.1–0.2. A portion (500 mL) of each culture was analyzed by western blotting using an anti-DnaA antibody **(D)** or an anti-DnaB **(E)** antibody. The indicated amounts of purified DnaA or DnaB were mixed with whole cell extract of NY26 (Δ*dnaA*) or ME5491 (*dnaB43*[Ts]) and the mixtures were used as a quantitative standard. ME5491 cells were grown at 30°C and further incubated for 3 h at 42°C. After precipitation with trichloroacetic acid, proteins were solubilized in SDS-sample buffer. The amounts of cellular DnaA or DnaB are indicated below the image.

To determine whether the suppression of *hda-185* by *yfdQRS* and *yfdQRT* (*yfdQRST*) was caused by inhibition of chromosomal replication overinitiation, we performed flow cytometry analysis (**Figure 2A**). Cells were grown at 42°C and incubated for five generations at 25°C, followed by further incubation in the presence of rifampicin and cephalexin, which inhibit the initiation replication and cell division, resulting in run-out replication of the chromosomes in each cell, as previously described (Skarstad et al., 1995). The number of chromosomes in the resultant cells corresponds to the number of sister *oriC* copies at the time of antibiotic addition to the growing cells. YH013 (*hda*-*185*) and YH014 (wild-type) cells bearing pBR322, pSSPB (pBR322-*yfdQRT*), or pSSPT (pBR322-*yfdQRS*) grew at 42°C in LB medium at similar rates (i.e., generation time 19–24 min) (**Table 2**). In M9 medium, growth of YH013 (*hda*-*185*) cells was severely inhibited, even at 42°C.

**Table 2:** Generation time of cells used for flow cytometry analysis.

Strain	Relevant genotype	Temperature (°C)	Medium	Plasmid	Generation time (min)
YH013	*hda-185*	42	LB	pBR322	20
				pSSPB	22
				pSSPT	24

YH014	wild-type	42	LB	pBR322	19
	*hda*			pSSPB	21
				pSSPT	19

MG1655	wild-type	37	LB	pBR322	21
				pSSPB	20
				pSSPT	21
			
			M9	pBR322	32
				pSSPB	34
				pSSPT	39

MIT147	Δ*seqA*	37	LB	pBR322	36
				pSSPB	30
				pSSPT	34
			
			M9	pBR322	38
				pSSPB	42
				pSSPT	42

SA103	Δ*diaA*	37	LB	pBR322	19
				pSSPB	26
				pSSPT	23
			
			M9	pBR322	31
				pSSPB	32
				pSSPT	32

MIT123	Δ*DARS1-2*	37	LB	pBR322	19
				pSSPB	23
				pSSPT	25
			
			M9	pBR322	32
				pSSPB	34
				pSSPT	38

NY5	Δ*yfdQRST*	37	LB	None	20
			
			M9	None	32


Flow cytometry data revealed that, when incubated at 25°C in LB medium, wild-type *hda* (YH014) cells bearing pBR322 contained four or eight chromosomes per cell, with four chromosomes predominating (**Figure 2A**). In *hda-185* (YH013) cells bearing pBR322, DNA content varied without yielding many discrete peaks and peaks equivalent to more than eight chromosomes were predominant, suggesting that severe overinitiation occurred and the replication forks were arrested on the way to the replication terminus (**Figure 2A**), as previously reported (Fujimitsu et al., 2008). In the *hda-185* (YH013) cells bearing pSSPB or pSSPT, discrete peaks corresponding to two to six chromosomes were evident, and four-chromosome population was predominant (**Figure 2A**). This indicated that pSSPB and pSSPT inhibited the overinitiation of chromosomal replication.

When the *hda-185* (YH013) cells bearing pBR322 were kept at 42°C in LB medium, DNA content varied and peaks equivalent to 7–8 chromosomes were predominant, suggesting that a moderate overinitiation occurred and the replication forks were arrested (**Figure 2A**). In the *hda-185* (YH013) cells bearing pSSPB or pSSPT, the overinitiation of chromosomal replication was inhibited. These results are consistent with those described above.

The cell mass of the *hda-185* (YH013) cells bearing pBR322 was greater than that of wild-type *hda* (YH014) cells bearing pBR322, indicating an inhibited cell division, as previously reported (Fujimitsu et al., 2008). When pSSPB or pSSPT were introduced into the *hda-185* (YH013) cells, the inhibition of cell division was repressed and the respective cell masses were similar to those of wild-type *hda* (YH014) cells bearing pSSPB or pSSPT. This can be linked to the inhibition of replication overinitiation (Fujimitsu et al., 2008).

In wild-type *hda* (YH014) cells bearing pSSPB or pSSPT, the predominant peak corresponded to four chromosomes, with the peaks of two and three chromosomes also evident (**Figure 2A**). The peak corresponding to eight chromosomes was small compared with that of wild-type *hda* (YH014) cells bearing pBR322. These results suggested that the increased copy number of *yfdQRST* inhibited the initiation even in wild-type cells. Similarly, as asynchronous initiations occurred, increased *yfdQRST* copy number interfered with regulatory mechanisms required for timely initiations at multiple *oriC* copies in a single cell.

### *yfdQRST* Inhibition of Replication Initiation Occurs in RIDA-Resistant *dnaA* Mutant Cells

To explore the mechanism of *hda-185* suppression by *yfdQRST*, we examined the effect of pSSPB and pSSPT in *dnaAcos* mutant cells. As described above, the initiation activity of DnaAcos is resistant to RIDA and is sustained over long periods at 30°C, resulting in overinitiation of replication and inhibition of colony formation (Katayama and Kornberg, 1994; Katayama, 1994; Chodavarapu et al., 2013). If the mechanism of *yfdQRST* initiation inhibition was independent of RIDA, the presence of pSSPB and pSSPT would suppress the defects of *dnaAcos* cells. On the other hand, if the suppression mechanism depended on augmentation of the activity of *hda-185* and RIDA, pSSPB and pSSPT would not suppress the defects of *dnaAcos* cells.

KH5402-1 (wild-type *dnaA*) and NA001 (*dnaAcos* derivative) cells bearing pBR322, pSSPB, or pSSPT were grown at 42°C and further incubated on LB agar plates at 42°C and 30°C to determine the colony-forming units (CFUs) (**Table 3**). The introduction of pSSPB and pSSPT did not affect the CFUs of KH5402-1 cells at either temperature (**Table 3**). CFUs of *dnaAcos* (NA001) cells bearing pBR322 were ∼10^5^-fold lower at 30°C than 42°C (**Table 3**), consistent with our previous study (Katayama and Kornberg, 1994). By contrast, CFUs of *dnaAcos* (NA001) cells bearing pSSPB or pSSPT were similar at 30°C and 42°C. These results indicated that pSSPB and pSSPT suppress the cold-sensitive colony formation of *dnaAcos* cells, in agreement with the data shown in **Figure 2A**. Also, these results are consistent with a RIDA-independent suppression exerted by *yfdQRST* increased copy number.

**Table 3:** pBR322-*yfdQRST* suppresses cold sensitivity of *dnaA*cos cells.

			CFU/mL (×10^8^)		
			
Strain	*dnaA*	Plasmid	30°C	42°C	30°C/42°C
KH5402-1	Wild-type	pBR322	7.6	7.6	1.0
		pSSPB	1.6	3.5	0.5
		pSSPT	2.5	3.4	0.7
NA001	*dnaAcos*	pBR322	8.5 × 10^-5^	4.0	2.1 × 10^-5^
		pSSPB	0.6	0.1	6.0
		pSSPT	0.3	1.0	0.3


*KH5402-1 (wild-type *dnaA*) or NA001 (*dnaAcos*) cells carrying pBR322, pSSPB, or pSSPT were grown at 42°C in LB medium containing 50 mg/mL ampicillin and 50 mg/mL thymine. Cells were serially diluted and incubated at 30°C for 30 h, or 42°C for 16 h, respectively, on LB agar plates containing 50 mg/mL thymine and 100 mg/mL ampicillin.*

### *yfdQRST* Inhibition of Replication Initiation Occurs in Δ*seqA* Cells

Next, we examined whether the suppression of *hda-185* observed after increasing *yfdQRST* copy number depends on SeqA, the *oriC*-binding inhibitor of initiation (Nievera et al., 2006; Waldminghaus and Skarstad, 2009). MG1655 and MIT147 (MG1655 Δ*seqA* derivative) cells bearing pBR322, pSSPB, or pSSPT were grown at 37°C in LB medium or M9 medium, and further incubated with rifampicin and cefalexin, followed by flow cytometry analysis (**Figures 2B,C**). The wild-type (MG1655) cells bearing either of the plasmids grew at comparable rates (i.e., generation time 32–39 min in M9 medium, and 20–21 min in LB medium) (**Table 2**). Likewise, the Δ*seqA* (MIT147) cells bearing either of the plasmids also grew at comparable rates (i.e., generation time 38–42 min in M9 medium, and 30–36 min in LB medium) (**Table 2**).

Whereas the wild-type (MG1655) cells bearing pBR322 contained two or four chromosomes per cell when grown in M9 medium, the majority of the Δ*seqA* (MIT147) cells bearing pBR322 contained DNA corresponding to four or more chromosomes (**Figure 2B**), indicating replication overinitiation, as reported previously (Lu et al., 1994). Introduction of pSSPB or pSSPT into the Δ*seqA* (MIT147) cells inhibited this overinitiation, as the majority of the cells contained a DNA equivalent of only two to four chromosomes. In addition, asynchronous initiations occurred even in the wild-type (MG1655) cells bearing pSSPB or pSSPT (**Figure 2B**). These results indicated that increasing the copy number of *yfdQRST* inhibits overinitiation in a SeqA-independent manner, and also inhibits the regulation of the timing of initiation, which was consistent with the results presented in **Figure 2A**. Cell mass of MG1655 cells bearing pSSPB or pSSPT was greater than that of MG1655 cells bearing pBR322 (**Figure 2B**), suggesting that overexpression of *yfdQRST* moderately inhibited cell division in M9 medium.

In LB medium, the wild-type (MG1655) cells bearing pBR322 contained eight chromosomes per cell, while the DNA content of the Δ*seqA* (MIT147) cells bearing pBR322 yielded no discrete peaks but some fractions equivalent to eight or more chromosomes predominated (**Figure 2C**), consistent with previous reports (Lu et al., 1994). Introduction of pSSPB or pSSPT into Δ*seqA* (MIT147) cells inhibited replication overinitiation, resulting in populations of cells bearing DNA content equivalent to eight or less chromosomes predominating. In addition, asynchronous initiations and inhibition of initiation occurred even in wild-type (MG1655) cells bearing pSSPB or pSSPT.

The introduction of pSSPB or pSSPT into MG1655 cells had little effect on cell division when the cells were grown in LB medium (**Figure 2C**). In addition, growth in LB medium might suppress synchronous initiations in these cells more effectively than in M9 medium (**Figures 2B,C**). Differences in protein expression between MG1655 cells growing in M9 and those growing in LB medium might affect YfdQRST functions and explain the differences observed using these two types of medium.

Furthermore, to determine whether pSSPB or pSSPT can inhibit expression of *dnaA* and *dnaB* genes, we investigated the amounts of DnaA and DnaB by immunoblot analysis. DnaA and DnaB protein levels in wild-type (MG1655) cells growing in LB medium were not appreciably affected by the introduction of the plasmids (**Figures 2D,E**).

### *yfdQRST* Inhibition of Replication Initiation Occurs in *DARS1*-*2* - Deleted Cells

**FIGURE 3:**
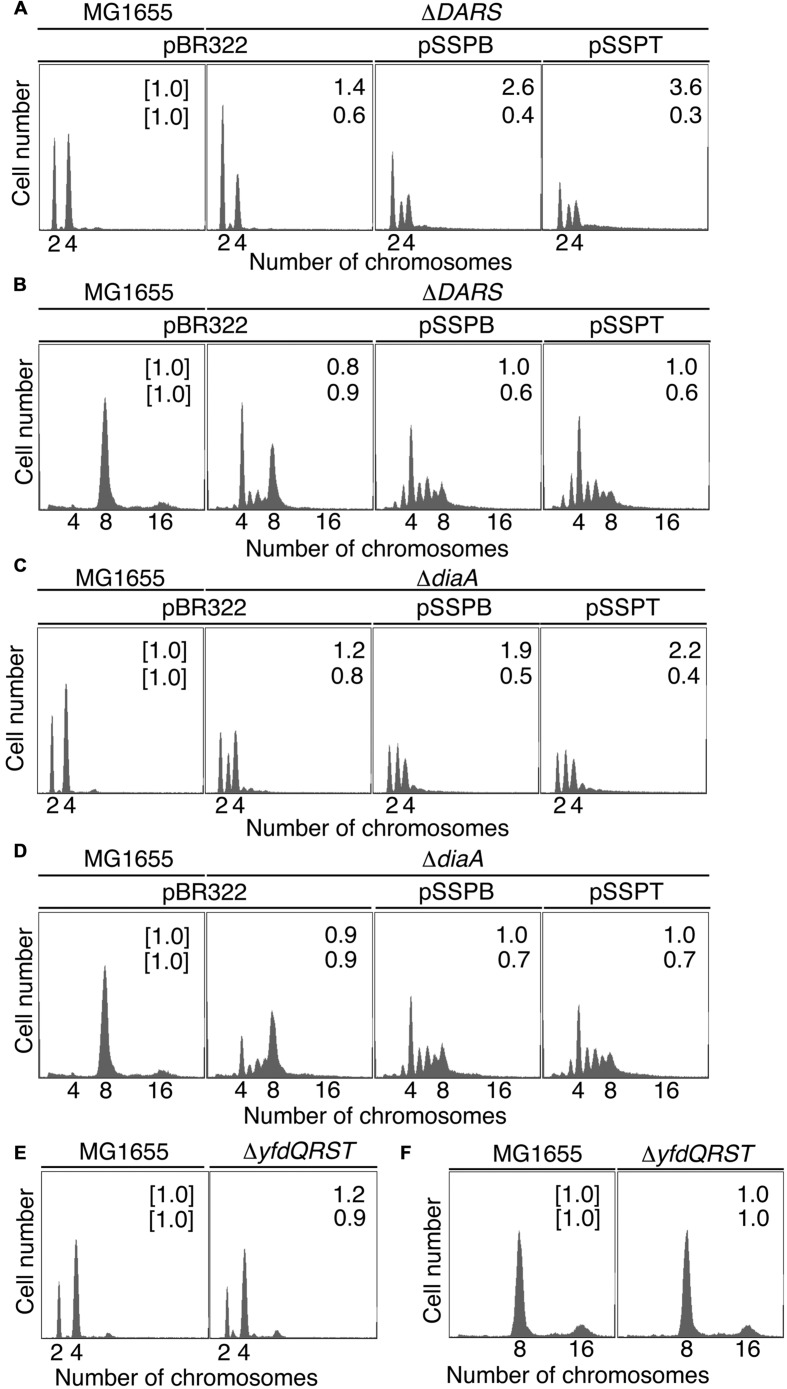
**pBR322-*yfdQRST* replication initiation inhibition in *diaA* and *DARS* mutants.** Cells bearing the indicated plasmids were grown at 37°C in M9 medium containing 0.2% glucose and 0.2% casamino acids **(A,C,E)** or LB medium **(B,D,F)**, and analyzed using flow cytometry, as described in **Figure 2B**. Mean cell masses (*upper*) and the *oriC* number per cell mass (*lower*) relative to MG1655 cells bearing pBR322 are indicated at the top right corners of each panel. **(A,B)** MG1655 and MIT123 (Δ*DARS*). **(C,D)** MG1655 and SA103 (Δ*diaA*). **(E,F)** MG1655 and NY5 (Δ*yfdQRST*).

Next, we examined whether suppressing *hda-185* by increasing *yfdQRST* copy numbers requires *DARS*. We analyzed MIT123 (MG1655 Δ*DARS1* Δ*DARS2* derivative) cells bearing pBR322, pSSPB, or pSSPT in M9 or LB medium using flow cytometry (**Figures 3A,B**). The Δ*DARS1* Δ*DARS2* (MIT123) cells bearing either of the plasmids grew at similar rates (i.e., generation time 32–38 min in M9 medium, and 19–25 min in LB medium, respectively) (**Table 2**). In M9 medium, the Δ*DARS1* Δ*DARS2* (MIT123) cells bearing pBR322 predominantly contained two chromosomes, compared with the wild-type (MG1655) cells bearing pBR322 (**Figure 3A**), indicating replication initiation inhibition, as reported previously (Fujimitsu et al., 2009). A considerable population of the Δ*DARS1* Δ*DARS2* (MIT123) cells bearing pSSPB or pSSPT contained three chromosomes (**Figure 3A**), indicating induction of asynchronous replication initiations. In LB medium, introduction of either pSSPB or pSSPT into the Δ*DARS1* Δ*DARS2* (MIT123) cells increased cell populations containing three or five to seven chromosomes, and decreased those containing eight chromosomes (**Figure 3B**). These results suggested that the increased copy number of *yfdQRST* inhibited replication initiation and also the regulation of initiation timing in a *DARS*-independent manner. This interpretation is consistent with a suppression mechanism that acts independently of the regulation of DnaA-nucleotide forms, and is consistent with the results shown in **Table 3**.

### *yfdQRST* Inhibition of Replication Initiation Occurs in Δ*diaA* Cells

We next examined whether suppressing *hda-185* by the increased *yfdQRST* copy number requires the function of DiaA. We analyzed SA103 (MG1655 Δ*diaA* derivative) cells bearing pBR322, pSSPB, or pSSPT in M9 or LB medium using flow cytometry (**Figures 3C,D**). If the suppression associated with elevated *yfdQRST* copy numbers requires DiaA, the peak pattern of DNA content in the Δ*diaA* (SA103) cells bearing pSSPB or pSSPT would be indistinguishable from that in the Δ*diaA* (SA103) cells bearing pBR322. On the other hand, if this effect can occur in the absence of DiaA, replication initiation would be inhibited in the Δ*diaA* (SA103) cells bearing pSSPB or pSSPT, with DNA peak patterns different from those of Δ*diaA* (SA103) cells bearing pBR322.

In M9 medium, Δ*diaA* (SA103) cells bearing pBR322, pSSPB, or pSSPT grew at the same rate (i.e., generation time 31–32 min) (**Table 2**). The wild-type (MG1655) cells bearing pBR322 contained two or four chromosomes, but Δ*diaA* deletion resulted in a decreased number of cells containing four chromosomes and an increased number of cells containing three chromosomes, accordingly (**Figure 3C**). This suggested the occurrence of initiation inhibition and asynchronous initiation, as reported previously (Ishida et al., 2004). Introduction of pSSPB or pSSPT into Δ*diaA* cells resulted in a slightly increased number of cells containing three chromosomes, and a decreased number of cells with four chromosomes, respectively (**Figure 3C**).

In LB medium, Δ*diaA* (SA103) cells bearing pBR322, pSSPB or pSSPT grew at comparable rates (i.e., generation time 19–26 min) (**Table 2**). Initiation inhibition occurred in the Δ*diaA* (SA103) cells bearing pBR322, and the introduction of pSSPB or pSSPT instead of pBR322 increased the occurrence of asynchronous initiation and further inhibited the initiation events (**Figure 3D**). These results indicated that increasing the *yfdQRST* copy numbers substantially inhibits replication initiation and the regulation of initiation timing even in the absence of DiaA. However, these results do not exclude the possibility that both DiaA-dependent and independent pathways function to inhibit replication initiation by *yfdQRST* (see below). In M9 medium, the initiation might be downregulated by multiple pathways and the inhibitory effect of YfdQRST might be relatively small.

### Increasing the *yfdQRST* Copy Number Affects the Activity of DnaA

To determine whether increasing the *yfdQRST* copy number affected the replication initiation activity of DnaA, we used a set of temperature-sensitive *dnaA* mutants (**Table 4**) (Hansen et al., 1992). These mutants are characterized by defective replication initiation and impaired colony formation at elevated temperatures. The *dnaA* mutant cells were transformed with pBR322, pSSPB, or pSSPT, and incubated at 30–42°C. When cells were transformed with pBR322, transformation efficiencies of all the strains were similar at 30°C (**Table 4**). However, when cells bearing the *dnaA5* or *dnaA204* allele were transformed with pSSPB or pSSPT, but not pBR322, colony formation was severely inhibited, even at 30°C (**Table 4**). In addition, when cells bearing the *dnaA46* or *dnaA604* allele were transformed with pSSPB or pSSPT, but not pBR322, colony formation was severely inhibited at 35°C, but not at 30°C. By contrast, transformation efficiencies with pSSPB or pSSPT of cells bearing the *dnaA508* or *dnaA167* allele were not substantially inhibited even at 37°C (**Table 4**). These results indicated that *yfdQRST*-dependent inhibition of colony formation occurs in a *dnaA* allele-specific manner and that the increased copy number of *yfdQRST* inhibits DnaA activity directly or indirectly. As *dnaA5* and *dnaA204* allele mutations are both located in domain IV of DnaA, *yfdQRST*-encoded proteins might primarily inhibit the process of DnaA-*oriC* complex formation. In addition, when *dnaA167* and *dnaA508* cells were analyzed using flow cytometry, *yfdQRST* inhibition of replication initiation was detected (**Supplementary Figure S1**), consistent with the data obtained with wild-type cells.

**Table 4:** pBR322-*yfdQRST* inhibits the growth of *dnaA*5 and *dnaA*204 cells at 30°C.

			Transformation efficiency (×10^5^)					
			
			30°C			35°C		
				
Strain	*dnaA*	Mutated domain	pBR322	pSSPB	pSSPT	pBR322	pSSPB	pSSPT
MG1655	+	None	1.4	0.8	1.1	1.3	1.0	1.1
MIT125	*46*	III	1.6	1.3	1.2	1.5	<4.0 × 10^-3^	<4.0 × 10^-3^
NY10	*5*	III, IV	1.8	<4.0 × 10^-3^	<4.0 × 10^-3^	1.6	<4.0 × 10^-3^	<4.0 × 10^-3^
NY11	*167*	III	1.6	1.0	1.0	1.3	1.1	1.1
NY12	*601*	III	1.1	1.0	1.0	0.9	<4.0 × 10^-3^	<4.0 × 10^-3^
MIT140	*508*	I	1.0	0.9	0.9	1.1	1.0	0.8
MIT143	*204*	IV	1.6	<4.0 × 10 ^-3^	<4.0 × 10^-3^	1.4	<4.0 × 10^-3^	<4.0 × 10^-3^

			**37°C**			**42°C**		
				
MG1655	+	None	1.3	1.1	1.5	1.5	0.9	1.2
MIT125	*46*	III	1.7	<4.0 × 10^-3^	<4.0 × 10^-3^	<4.0 × 10^-3^	<4.0 × 10^-3^	<4.0 × 10^-3^
NY10	*5*	III, IV	1.5	<4.0 × 10^-3^	<4.0 × 10^-3^	<4.0 × 10^-3^	<4.0 × 10^-3^	<4.0 × 10^-3^
NY11	*167*	III	1.3	1.2	1.1	<4.0 × 10^-3^	<4.0 × 10^-3^	<4.0 × 10^-3^
NY12	*601*	III	1.1	<4.0 × 10^-3^	<4.0 × 10^-3^	<4.0 × 10^-3^	<4.0 × 10^-3^	<4.0 × 10^-3^
MIT140	*508*	I	1.0	0.9	0.8	<4.0 × 10^-3^	<4.0 × 10^-3^	<4.0 × 10^-3^
MIT143	*204*	IV	1.5	<4.0 × 10^-3^	<4.0 × 10^-3^	<4.0 × 10^-3^	<4.0 × 10^-3^	<4.0 × 10^-3^


*MG1655 cells or a set of MG1655-derivative *dnaA* cells were transformed with pBR322, pSSPB, or pSSPT, and incubated at 30°C for 24 h, or 35°C, 37°C, and 42°C for 13 h, respectively, on LB agar plates containing 100 mg/mL ampicillin. Transformation efficiencies are shown. +, wild-type.*

To distinguish between the possibilities that pSSPB and pSSPT either induce severe replication overinitiation or inhibit the initiation in *dnaA5* and *dnaA204* cells, we examined plasmid transformation efficiencies in cells bearing *rnhA* mutation. Deletion of *rnhA* allows the activation of an alternative replication origin, a DnaA-independent initiation, and enables growth of DnaA-defective cells (Kogoma, 1997). When *rnhA*-defective cells bearing the *dnaA5* or *dnaA204* allele were transformed with pSSPB or pSSPT, the transformant colony formation was not inhibited (**Table 5**). These results indicated that the absence of *rnhA* rescued the growth of *dnaA5* and *dnaA204* mutants bearing pSSPB and pSSPT. This is consistent with the idea that the increased copy number of *yfdQRST* inhibits the initiation of replication by interacting with DnaA.

**Table 5:** Deletion of *rnhA* suppresses the growth inhibition of *dnaA5 and dnaA204* cells carrying pBR322-*yfdQRST.*

			Transformation efficiency (×10^5^)		
			
Strain	*dnaA*	*rnhA*	pBR322	pSSPB	pSSPT
MG1655	+	+	1.1	0.9	1.0
MIT162	+	::*cat*	6.2	3.1	3.5
NY10	*5*	+	1.5	4.0 × 10^-2^	1.0 × 10^-2^
NY16	*5*	::*cat*	2.5	1.3	1.4
MIT143	*204*	+	2.4	1.0 × 10^-2^	<1.0 × 10^-2^
NY17	*204*	::*cat*	0.5	0.3	0.2


*MG1655 and its derivative cells bearing *dnaA5, dnaA204 or/and rnhA*::*cat* were transformed with pBR322, pSSPB, or pSSPT, and incubated at 30 °C for 30 h on LB agar plates containing 100 mg/mL ampicillin. Transformation efficiencies are shown. +, wild-type.*

### Initiation of Chromosomal Replication in *yfdQRST*-Deletion Mutant

We next used flow cytometry to investigate the replication initiation activity in *yfdQRST*-deletion mutants. MG1655 chromosomal *yfdQRST* region was replaced with *kan* gene using λRed system (Datsenko and Wanner, 2000), and the resultant strain was NY5. MG1655 and NY5 (Δ*yfdQRST*) cells were grown in M9 medium, or LB medium, at 37°C and then analyzed by flow cytometry (**Figures 3E,F**). Both strains grew at the same rates under these conditions (i.e., generation time 32 min in M9 medium, and 20 min in LB medium) (**Table 2**). The DNA content in MG1655 and NY5 (Δ*yfdQRST*) cells was virtually indistinguishable after growth in the two media. Therefore, the function of *yfdQRST* might be redundant with that of other bacterial genes, or might be important only under certain conditions, e.g., specific environmental stresses (see Discussion).

### HU Sensitivity

**FIGURE 4:**
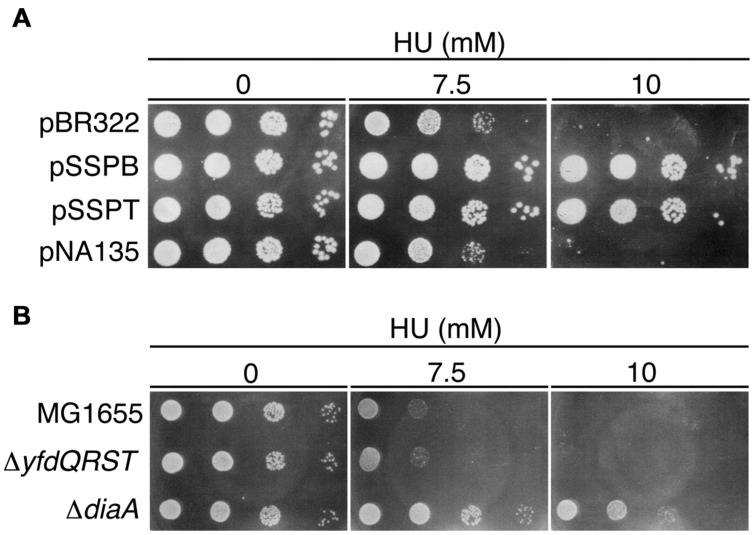
**Hydroxyurea (HU) resistance of cells carrying pBR322-*yfdQRST*.** **(A)** MG1655 cells bearing pBR322, pSSPB, pSSPT, or pNA135 (pBR322 derivatives carrying *diaA*) were incubated at 37°C overnight, serially diluted, and spotted on LB plates containing the indicated amount of HU, and further incubated at 37°C. **(B)** MG1655, NY5 (Δ*yfdQRST*), or SA103 (Δ*diaA*), analyzed as described in **(A)**.

Colony formation of the *seqA* mutant is hypersensitive to HU, which depletes intracellular dNTPs pools and causes replication fork arrest (Sutera and Lovett, 2006). This hypersensitivity is suppressed by the introduction of a temperature-sensitive *dnaA46* mutation, which causes a reduced replication initiation even at 30°C. Indeed, it is suggested that the reduced replication initiation causes the resistance to HU (Sutera and Lovett, 2006). Accordingly, we assessed the HU sensitivity of cells bearing pSSPB or pSSPT. The growth of MG1655 cells bearing pBR322 was severely inhibited in the presence of 10 mM HU. By contrast, cells bearing pSSPB or pSSPT were HU-resistant (**Figure 4A**).

HU sensitivity of Δ*yfdQRST* (NY5) cells was similar to that of MG1655 cells (**Figure 4B**), consistent with our findings that replication initiation in these cells is not significantly affected (**Figures 3E,F**). We assessed HU sensitivity of *diaA* mutants in a similar manner. Both *diaA* excess and deficiency suppress the cold sensitivity of colony formation of *hda*-*185* cells (Fujimitsu et al., 2008). HU sensitivity of MG1655 cells bearing a multicopy plasmid carrying *diaA* (pNA135) was similar to that of cells bearing pBR322 (**Figure 4A**). By contrast, *diaA* mutant cells were HU-resistant (**Figure 4B**). This suggested that the inhibitory mechanism of replication initiation associated with increased *yfdQRST* copy number is similar to the effect of *diaA* deletion rather than *diaA* excess (see Discussion).

### YfdR Binds DnaA

**FIGURE 5:**
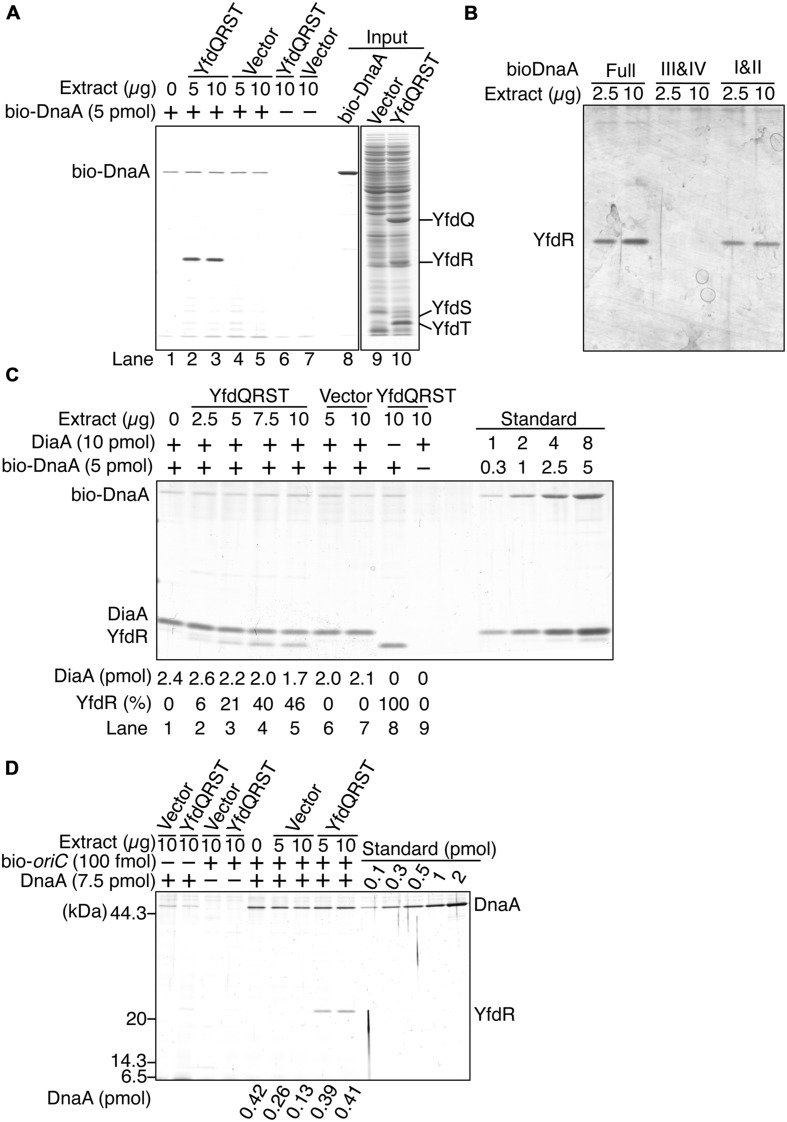
**DnaA binds YfdR in protein extracts.** **(A)** The indicated amounts of MG1655 protein extracts from strains carrying pQRST (YfdQRST) or pBAD18 (Vector) were incubated in the presence or absence of bio-DnaA. Proteins bound to DnaA were isolated using streptavidin beads and analyzed by SDS-PAGE and silver staining. The amount of proteins in crude fractions was analyzed using Coomassie Brilliant Blue assay. Final gel positions of YfdQ, YfdR, YfdS, and YfdT, as expected from the calculated molecular sizes, are indicated. bio-DnaA remained bound to streptavidin beads, and, consequently, only weak DnaA bands were detected. **(B)** The indicated amounts of proteins from extracts from MG1655 cells carrying pQRST were incubated in a buffer containing bio-DnaA (Full) or its truncated forms either bearing domain I and II (I&II), or domain III and IV (III&IV). Proteins bound to DnaA were isolated using streptavidin beads and analyzed by SDS-PAGE and silver staining. Bands corresponding to YfdR are indicated. **(C)** The indicated amounts of proteins from extracts from MG1655 cells carrying pQRST (YfdQRST) or pBAD18 (Vector) were incubated in the presence or absence of bio-DnaA (5 pmol) or DiaA (10 pmol). Proteins bound to DnaA were isolated using streptavidin beads and analyzed by SDS-PAGE and silver staining. bio-DnaA remained bound to streptavidin beads, and therefore only weak DnaA bands were detected. DiaA amounts were determined using a standard curve and are indicated below the gel image. The relative intensities of YfdR were determined as a ratio to the intensity in lane 8, and are also indicated below the gel image. **(D)** The indicated amounts of proteins in extracts of MG1655 cells carrying pQRST (YfdQRST) or pBAD18 (Vector) were incubated in the presence or absence of DnaA (7.5 pmol) or a biotin-tagged *oriC* fragment (bio-*oriC*) (100 fmol). Proteins bound to bio-*oriC* were pulled down with streptavidin beads and analyzed by SDS-PAGE and silver staining. DnaA amounts were determined using a standard curve and are indicated below the gel image. The amounts of DnaA are given after subtraction of the amount of bead-bound DnaA.

To investigate whether YfdQ, YfdR, YfdS, and YfdT proteins (YfdQRST) bind DnaA, we performed a pull-down assay using bio-DnaA and protein extracts from MG1655 cells carrying either an empty vector pBAD18 or the YfdQRST-overproducing plasmid pQRST. YfdQ, YfdR, YfdS, and YfdT proteins were concentrated in the protein extract of pQRST-bearing cells, and formed prominent bands in SDS-polyacrylamide gel (**Figure 5A**, lane 10). bio-DnaA was incubated on ice in a buffer containing the protein extract. DnaA-bound proteins were recovered using streptavidin-conjugated beads, eluted into SDS, and analyzed with SDS-PAGE. The results indicated that only YfdR bound DnaA (**Figure 5A**). Most DnaA molecules remained bound to the beads because of the tight binding of biotin to streptavidin.

We investigated the YfdR-binding region of DnaA using biotin-tagged truncated DnaA protein versions, consisting of domains I–II, or domains III–IV (Ishida et al., 2004). Pull-down assays revealed that YfdR bound DnaA domains I–II, but not domains III–IV (**Figure 5B**). DiaA also binds DnaA in Domain I, in a Phe46-dependent manner (Keyamura et al., 2009). YfdR-DnaA binding could competitively inhibit DiaA-DnaA binding. To evaluate whether YfdR competes for DnaA binding with DiaA, we performed similar pull-down assays using purified DiaA (**Figure 5C**). bio-DnaA was first incubated with DiaA, following which the mixture was further incubated with the protein extracts. The binding of YfdR to DnaA was lower in the presence of DiaA than in the absence of DiaA (**Figure 5C**, lanes 5 and 8). The binding of DiaA to DnaA was slightly lower when excessive amounts of protein extracts from cells carrying pQRST were incubated with the DiaA-DnaA complex (**Figure 5C**, lanes 5 and 7). These results are basically consistent with the data presented in **Figure 5B**.

Furthermore, we investigated whether YfdR binds to DnaA complexes constructed on *oriC*, by using a pull-down assay with a biotin-tagged *oriC* DNA fragment (bio-*oriC*) (Keyamura et al., 2007, 2009; Ozaki and Katayama, 2012; Ozaki et al., 2012). DnaA was first incubated with bio-*oriC* and then with protein extracts, and the *oriC*-bound proteins were recovered using streptavidin-conjugated beads (**Figure 5D**). When bio-*oriC* was absent, only faint bands corresponding to DnaA were detected, which were probably the result of non-specific binding of DnaA to the beads. The intensities of these bands were quantified and subtracted from those of other DnaA bands to control for this background level of DnaA binding to the beads. In the presence of bio-*oriC*, recovered DnaA amounts were increased. YfdR and DnaA were recovered when the extract from cells bearing pQRST was incubated with DnaA and bio-*oriC*, but not when the extract from cells bearing pBAD18 (vector) was employed. This is entirely consistent with the data shown in **Figure 5A**. DnaA protein recovery was moderately reduced when the extract from cells bearing pBAD18 was used but not when the extract from cells bearing pQRST was used. Binding of YfdR to DnaA might change the conformation of DnaA to make it resistant to possible inhibitors in the extract. Recovery of YfdQRST was not substantially detected when DnaA was not added to the assay, suggesting that YfdQRST does not bind to *oriC* DNA stably at least under these conditions (**Figure 5D**).

### DnaA-Binding Specificity of YfdR

**FIGURE 6:**
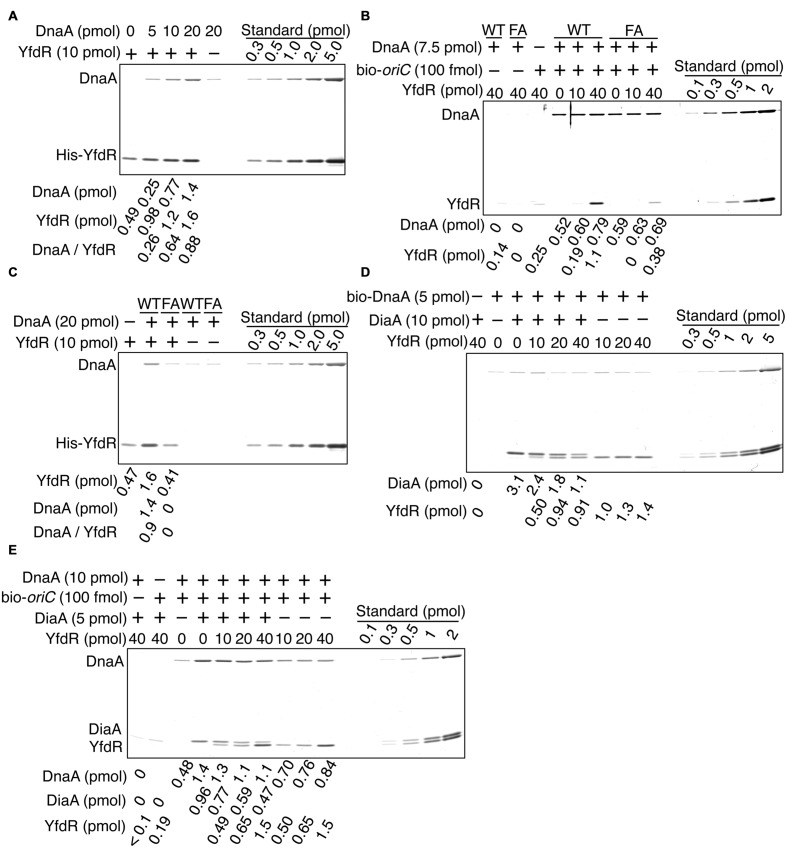
**DnaA binds to purified YfdR.** **(A)** His-YfdR (10 pmol) was incubated for 15 min on ice in a buffer containing the indicated amounts of DnaA. Proteins bound to His-YfdR were isolated using Co^2+^-conjugated beads (Dynabeads) and analyzed by SDS-PAGE and silver staining. The amounts of His-YfdR and DnaA were determined using a standard curve and are indicated below the gel image. The amounts of DnaA are given after subtraction of the amount of bead-bound DnaA **(B)** The indicated amounts of His-YfdR were incubated for 15 min on ice in a buffer containing bio-*oriC* (100 fmol) and wild-type DnaA (WT) or DnaA F46A (FA) (7.5 pmol). Proteins bound to bio-*oriC* were pulled down with streptavidin beads and analyzed by SDS-PAGE and silver staining. The amounts of His-YfdR and DnaA were determined using a standard curve and are indicated below the gel image. **(C)** His-YfdR (10 pmol) was incubated for 15 min on ice in a buffer containing intact DnaA (WT) or DnaA F46A (FA) (20 pmol). Proteins bound to His-YfdR were isolated using Dynabeads and analyzed by SDS-PAGE and silver staining. The amounts of His-YfdR and DnaA were determined using a standard curve and are indicated below the gel image. The amounts of DnaA are given after subtraction of the amount of bead-bound DnaA. **(D)** DiaA (10 pmol as monomer) was incubated for 5 min on ice in a buffer containing bio-DnaA (5 pmol). The mixture was further incubated with the indicated amounts of His-YfdR for 10 min. Proteins bound to DnaA were analyzed as described in the **Figure 5** legend. The amounts of His-YfdR, DiaA and DnaA were determined using a standard curve and are indicated as monomer below the gel image. **(E)** DiaA (10 pmol as monomer) was incubated for 5 min on ice in a buffer containing bio-*oriC* (100 fmol) and DnaA (7.5 pmol). The mixture was further incubated with the indicated amounts of His-YfdR for 10 min. Proteins bound to bio-*oriC* were analyzed as described above. The amounts (pmol) of His-YfdR, DiaA and DnaA were determined using a standard curve and are indicated as monomer below the gel image.

To determine whether YfdR binds DnaA directly, we purified His-tagged YfdR and performed a pull-down assay. His-YfdR was incubated in a buffer containing native DnaA, and proteins bound to YfdR were recovered using Co^2+^-conjugated beads. DnaA protein has a strong propensity to bind the beads non-specifically, especially when the imidazole concentration is low. Thus, in these pull-down experiments, imidazole was added at a relatively high concentration to the binding and wash buffers (i.e., 20 mM and 50 mM, respectively). Although this caused a reduction in the yield of His-YfdR, DnaA was recovered in a YfdR-dependent manner, with stoichiometric binding efficiency (**Figure 6A**). The slight level of non-specific DnaA binding to the beads was subtracted from those of other assays to control for this background level of DnaA binding. These data are consistent with the results of the pull-down assays with protein extracts and the results of *in vivo* analyses. In addition, the recovery of YfdR increased in a DnaA dose-dependent manner (**Figure 6A**). When a single YfdR molecule binds DnaA, additional YfdR molecules might bind to the complex and form YfdR multimers, or, alternatively, the YfdR conformation might change, increasing its affinity for Co^2+^ beads. Taken together, these results revealed that YfdR is a novel DnaA-binding protein.

DiaA and DnaB specifically bind DnaA in domain I carrying Phe46 residue (Abe et al., 2007; Keyamura et al., 2009). To investigate whether YfdR binding to DnaA also depends on Phe46, we performed pull-down assays with a DnaA F46A mutant protein bearing substitution of Phe46 with Ala residue. In our previous study, purified DnaA F46A retained ATP binding, *oriC* binding, and *oriC* unwinding activities at levels similar to those of wild-type DnaA (Keyamura et al., 2009). Similarly, in the present study, DnaA F46A pulled-down using biotin-tagged *oriC* DNA was as active for *oriC* binding as wild-type DnaA (**Figure 6B**). Next, we investigated whether DnaA F46A binds YfdR using a pull-down assay with bio-*oriC* (**Figure 6B**). Although YfdR showed only a slight non-specific binding to streptavidine-beads, the protein was efficiently recovered when both wild-type DnaA and bio-*oriC* were co-incubated. These results indicate that even when DnaA is complexed to *oriC* it can bind YfdR with stoichiometric binding efficiency. However, when DnaA F46A was used, recovery of YfdR was less efficient.

Next, we performed a pull-down assay with His-YfdR and wild-type DnaA or DnaA F46A in the absence of *oriC* (**Figure 6C**). As observed in **Figure 6A**, slight binding of DnaA to the beads was detected even in the absence of His-YfdR. This background level of binding was quantified and subtracted from the quantified levels of other DnaA bands to control for this background level of DnaA binding. The results indicated that the binding of YfdR to DnaA F46A was weak. As shown in **Figure 6A**, His-YfdR binding to the beads was increased when wild-type DnaA was co-incubated with His-YfdR (**Figure 6C**), further confirming the conjecture that DnaA binding to His-YfdR can change the conformation of YfdR to increase its stable binding to Co^2+^-beads. This conformational change could involve the formation of YfdR oligomers. YfdR recovery did not improve in the presence of DnaA F46A, supporting the idea that YfdR does not bind DnaA F46A (**Figure 6C**). In the presence of *oriC*, DnaA multimerizes, which might result in the formation of a second YfdR-binding site on the surface of DnaA complexes and explain the residual binding of DnaA F46A to YfdR (**Figure 6B**). Thus, these results suggest that DnaA Phe46 plays an important role in DnaA-YfdR binding and are consistent with the data shown in **Figure 5**.

### YfdR Inhibition of DiaA Binding to DnaA Complexes Formed on *oriC*

Here, we investigated whether purified His-YfdR inhibits DiaA binding to DnaA. Bio-DnaA was incubated with DiaA, followed by further incubation with His-YfdR and protein recovery using streptavidin-beads. Addition of His-YfdR moderately decreased the binding of DiaA to DnaA, suggesting moderate competition between DiaA and YfdR for binding to DnaA (**Figure 6D**). YfdR inhibition of DiaA-DnaA binding was increased when purified His-YfdR was used (**Figures 5C** and **6D**).

Next, we employed a pull-down assay using bio-*oriC* to investigate whether YfdR inhibits DiaA binding to DnaA complexes on *oriC*. DnaA was incubated with bio-*oriC* and DiaA, followed by further incubation with His-YfdR (**Figure 6E**). The recovery of DnaA was quantitatively analyzed as described in the legend of **Figure 5D**. DiaA increased DnaA binding to *oriC* and DiaA bound to DnaA complexed with *oriC* with stoichiometric efficiency. As DiaA forms homotetramers, two or three DiaA tetramers bound to a single *oriC*-DnaA complex. These results are consistent with our previous results (Keyamura et al., 2007, 2009). Addition of His-YfdR moderately decreased the binding of DiaA to DnaA bound to *oriC*. These results suggest that DiaA and YfdR moderately compete for binding to DnaA. It is possible that a single *oriC*-DnaA complex can retain both DiaA and YfdR at the same time. Alternatively, mixtures composed of *oriC*-DnaA complexes bound only to DiaA or only to YfdR may exist.

### YfdR Inhibition of DnaB Binding to DnaA Complexes Formed on *oriC*

DnaB binds to DnaA in a domain I Phe46-dependent manner with a weak affinity (Sutton et al., 1998; Abe et al., 2007; Keyamura et al., 2009). DnaB binding to DnaA oligomers formed on *oriC*, but not *oriC*-free DnaA monomers, can be detected by a pull-down assay. As DnaB forms stable homohexamers, a DnaA oligomer bound to *oriC*, but not free DnaA monomers, can provide a DnaB homohexamer with multiple binding points, thereby resulting in an increase of overall affinity (Stauffer and Chazin, 2004; Abe et al., 2007; Keyamura et al., 2009).

**FIGURE 7:**
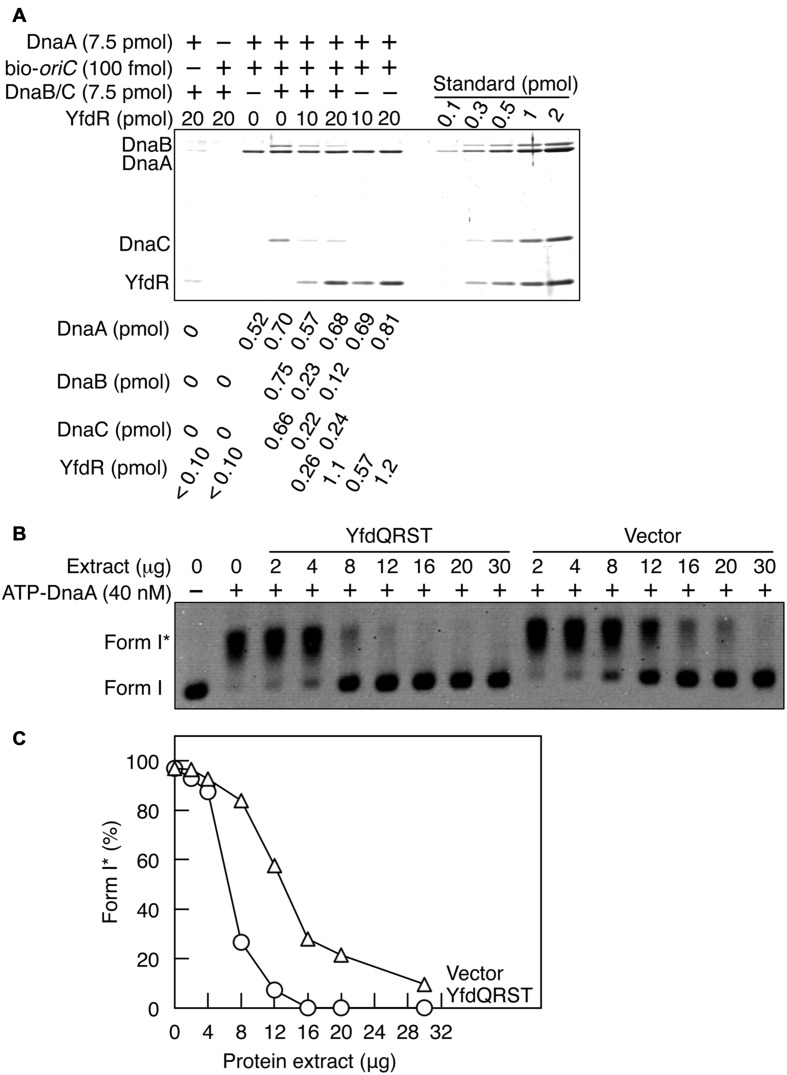
**YfdQRST inhibits the initiation of replication *in vitro*.** **(A)** DnaB (7.5 pmol as monomer) and DnaC (7.5 pmol) were co-incubated on ice, added to a buffer containing bio-*oriC* (100 fmol) and DnaA (7.5 pmol), and incubated for 5 min on ice. The mixture was further incubated with the indicated amounts of His-YfdR for 10 min. Proteins bound to bio-*oriC* were pulled down with streptavidin beads and analyzed by SDS-PAGE and silver staining. The amounts of His-YfdR, DnaB, DnaC, and DnaA were determined using a standard curve and are indicated below the gel image. **(B,C)** The indicated amounts of proteins from extracts of MG1655 cells carrying pQRST (YfdQRST) or pBAD18 (Vector) were incubated on ice in a buffer containing pBRoriC (1.6 nM, as plasmid), DnaB helicase, DnaC helicase loader, IHF, SSB, and gyrase. The mixture was further incubated with ATP-DnaA (40 nM) for 15 min at 30°C. Reactions were assessed using agarose gel electrophoresis **(B)**. Gel image is shown in the black/white inverted mode, and the migration positions of form I and form I^∗^ DNAs are indicated. The relative amounts of form I^∗^ are presented as ratios to total DNA (%) **(C)**.

Here we employed a pull-down assay using bio-*oriC* to investigate whether YfdR inhibits DnaB binding to DnaA complexes on *oriC*. DnaA was incubated with bio-*oriC* and DnaB-DnaC complexes, followed by further incubation with His-YfdR (**Figure 7A**). DnaC-helicase loader forms a stable complex with a DnaB hexamer. The recovery of DnaA was quantitatively analyzed as described for **Figure 5D**. Addition of DnaB-DnaC complexes slightly increased the DnaA recovery. One or two DnaB-DnaC complexes were shown to bind to a single *oriC*-DnaA complex. There results are well consistent with our previous results (Ozaki and Katayama, 2012). Addition of His-YfdR moderately decreased the binding levels of DnaB-DnaC complexes to DnaA bound to *oriC*. A slight residual level of DnaC could be due to possible, weak interaction of DnaC with DnaA-YfdR complexes constructed on *oriC*. When 20 pmol YfdR were incubated, a slight, non-specific recovery was detected but the recovery of the protein was efficient in the presence of both DnaA and bio-*oriC*. These results suggest that DnaB and YfdR basically compete for binding to DnaA. This is consistent also with our previous results that affinity of DnaA for DnaB is lower than that for DiaA (Keyamura et al., 2009).

### YfdQRST Proteins Inhibit Chromosomal Replication Initiation in an *oriC*-Dependent Replication Initiation System *In Vitro*

Next, we assessed whether the YfdQRST proteins inhibit replication initiation *in vitro* using a form I^∗^ assay, which assesses *oriC* unwinding and DnaB loading. In this assay, if DnaB is loaded onto unwound DNA region of the *oriC* plasmid supercoiled form (form I) and its helicase activity causes the ssDNA region to expand, the gyrase introduces further superhelicity, resulting in a highly negative supercoiled form (form I^∗^). Form I and form I^∗^ of the plasmid can be distinguished by agarose gel electrophoresis.

Protein extracts used in **Figure 5** were incubated with pBRoriC form I, IHF, DnaB, DnaC, SSB, and gyrase, on ice, followed by further incubation with ATP-DnaA at 30°C (**Figures 7B,C**). Form I^∗^ was efficiently produced in the absence of protein extracts (**Figures 7B,C**). The protein extract from cells carrying pQRST was more inhibitory to the production of form I^∗^ than that from cells carrying pBAD18 (**Figures 7B,C**). Excessive amounts of pBAD18-bearing cell extracts impaired the production of form I^∗^, which could result from non-specific inhibitors present in these protein extracts. These results suggested that YfdQRST proteins inhibit replication initiation at *oriC in vitro*, which was consistent with *in vivo* results described above. When purified YfdR was used in this assay instead of the YfdQRST extract, form I^∗^ production was not significantly inhibited (data not shown). This is consistent with our *in vivo* data, which revealed that the *yfdQRST* gene set was required for the suppression of *hda-185* mutation. Initiation inhibition by YfdQRST, therefore, requires other specific functions of YfdQST proteins *in vivo*, in addition to YfdR binding DnaA. Although purified YfdR inhibited DnaA-DnaB interaction in the pull-down assay, it is possible that a certain protein included in the form I^∗^ assay, in addition to DnaA, interacts with YfdR and reduces the inhibitory effect and that YfdQST enhances YfdR inhibition to DnaA-DnaB interaction under these conditions (see Discussion).

## Discussion

In this study, we isolated the multicopy suppressors of *hda-185* mutation and discovered that *yfdQRST* gene cluster of a cryptic phage CPS-53 was responsible for the suppression. The suppression was caused by the inhibition of *hda-185-*dependent replication overinitiation. In addition, the increased *yfdQRST* copy numbers inhibited the overinitiation in *dnaAcos* and *seqA* mutants, as well as appropriate initiation in wild-type cells. Furthermore, increasing the copies of *yfdQRST* led to inhibited colony formation of temperature-sensitive *dnaA* cells with a mutation in DnaA domain IV, at 30°C. These results are consistent with the hypothesis that higher *yfdQRST* copy numbers inhibit the initiation at *oriC* through interfering with DnaA function. Moreover, the *in vitro* experiments demonstrated that YfdR binds DnaA directly, DnaA Phe46 plays an important role in YfdR-DnaA binding, and YfdR moderately inhibits DiaA-DnaA binding and DnaB-DnaA binding. Further *in vitro* experiments suggested that YfdQRST proteins inhibit replication initiation, supporting our initial interpretations of the *in vivo* data. Taken together, we propose that YfdR is a novel DnaA-binding protein, and an idea that YfdQRST concordantly plays a role in inhibiting the initiation of chromosomal replication under specific stress conditions under which *yfdQRST* expression is increased. Deletion of *yfdQRST* did not affect replication initiation under normal growth conditions. Since the replication initiation is tightly regulated by multiple and redundant mechanisms, the involvement of *yfdQRST* in this process might be dispensable under normal conditions. In addition, we cannot exclude the possibility that the effect of YfdQRST overexpression might be an artifact and might not reflect the true role of this protein.

YfdQRST-dependent inhibition of replication initiation occurred in the absence of the functions of RIDA, *DARS*, SeqA, and DiaA (**Figures 2** and **3**; **Table 3**). This suggests that YfdQRST may inhibit initiation via a novel mechanism(s). In addition, as described above, DnaA Phe46 is important for YfdR-DnaA binding and YfdR moderately competes with DiaA and DnaB for DnaA binding (**Figures 5C**, **6** and **7A**). There is evidence to indicate that DnaA Phe46 acts as a specific and common binding site for the binding of DiaA and DnaB to DnaA (Abe et al., 2007; Keyamura et al., 2009). Thus, at least two non-mutually exclusive mechanisms are conceivable for the YfdQRST mode of inhibition: (1) inhibition of the DnaA-DiaA interaction, resulting in the inhibition of DnaA assembly and *oriC* unwinding, and (2) inhibition of the DnaA-DnaB interaction, resulting in failure of DnaB to load onto ssDNA. In the absence of DiaA, the second mechanism of inhibition of the DnaA-DnaB interaction might function effectively alone. When the DnaA–DiaA interaction is inhibited in wild-type *diaA* cells by competitive binding of YfdR, DnaA assembly might be impaired, resulting in the inhibition of replication initiation, as in the case of *diaA*-null mutant cells (Ishida et al., 2004; Keyamura et al., 2007). This assumption is consistent with flow cytometry analysis data, and the HU resistance (**Figure 4**) of YfdQRST-overproducing cells and *diaA*-null mutant cells. In addition, it is possible that other mechanisms exist in addition to the above two. For example, although YfdQRST do not tightly bind to *oriC in vitro*, those or one of those could bind to *oriC* under the *in vivo* conditions.

We previously reported that the *diaA*-null mutation inhibits colony formation of *dnaA5*, *dnaA46*, and *dnaA601* cells at semi-permissive temperatures, i.e., 35–37°C (Ishida et al., 2004). In addition, the growth of cells bearing Δ*diaA dnaA204* double mutations was also inhibited at 30°C, resulting in heterologous colony sizes (data not shown). Overproduction of YfdQRST inhibited colony formation by *dnaA5*, *dnaA46*, *dnaA601*, and *dnaA204* cells at 35°C or 30°C, similarly to the disruption of *diaA* in those cells (**Table 4**). A basic mechanism for YfdQRST-dependent inhibition of replication initiation is likely to mimic *diaA* disruption. Also, considering that the overproduction of YfdQRST inhibited colony formation of *dnaA5* and *dnaA204* cells even at 30°C, that the effect was more severe than *diaA* disruption in those cells (**Table 4**), and that the introduction of multiple copies of *yfdQRST* into *diaA* mutant cells increased asynchronous initiation and inhibition of initiation (**Figures 3C,D**), the inhibition of the DiaA–DnaA interaction is likely not the only mechanism of YfdQRST-dependent replication initiation inhibition, which is consistent with the aforementioned idea that two inhibitory mechanisms, i.e., inhibitions of DiaA–DnaA and DnaB–DnaA interactions, function.

In addition to the inhibition of the DnaA–DiaA interaction, it is possible that YfdQRST also inhibits the DnaA–DnaB interaction. This is based on the data indicating the competition of YfdR with DnaB for DnaA binding (**Figure 7A**) and would explain the inhibition of replication initiation in an *in vitro* reconstituted system (**Figures 7B,C**), and the *in vivo* data (**Figures 2** and **3**; **Tables 3**–**5**) except for HU resistance (**Figure 4**). The data on HU resistance might reflect the inhibition of DnaA assembly, which precedes DnaB loading. It is conceivable that when the both inhibition mechanisms occur in YfdQRST-overproducing cells, complex phenotypes can result.

The roles for YfdQST proteins (excluding YfdR) remain unclear. The pull-down experiments suggest that YfdQST do not possess high affinities for either YfdR or DnaA (**Figure 5A**). However, it is not unfeasible that an unstable interaction of these proteins with either YdfR or DnaA could stimulate YfdR–DnaA binding through conformational changes of the proteins. In addition, YfdQST could interact with DiaA and thereby change its conformation, reducing its affinity for DnaA. Similarly, YfdQST could interact with DnaB and thereby change its conformation, reducing its affinity for DnaA. These interactions and associated conformational changes could enhance the interaction between YfdR and DnaA, and thereby inhibit initiation *in vivo*. The flow cytometry data (**Figures 2** and **3**) suggested that pSSPT was slightly more effective in inhibiting overinitiation than pSSPB, consistent with an idea that YfdS might be more effective than YfdT. Further detailed analyses will be required to elucidate these mechanisms.

YfdQRST are encoded by a set of genes of the cryptic phage CPS-53. These proteins could have played an important role for phage growth in the ancestral CPS-53 propagative phases. λ phage P protein plays a crucial role in the replication of phage DNA, while it inhibits the initiation of host chromosome replication by binding to the DnaA domain III (Datta et al., 2005). λ phage-encoded O and P proteins form heterocomplexes on the cognate origin, and the P protein binds DnaB helicase to recruit it. Also, P protein binds to the DnaA domain III, inhibiting ATP binding and cooperative DnaA binding to *oriC* (Datta et al., 2005). This inhibition supposedly enhances phage DNA replication and lethality to the host. Replication inhibition by YfdQRST may have played a similar role in the evolutionarily ancient version of CPS-53 which was propagative phage and might be absent from present day *E. coli* cells. In this context, it is notable that ORFs29-31 in the genome of *Shigella flexneri* bacteriophage V encode YfdQRST homologues (Allison et al., 2002).

The *E. coli* chromosome contains nine cryptic prophages (Wang et al., 2010). A recent report revealed, through deletion analysis, that some of these prophages assist the host survival amid adverse environmental factors, such as H_2_O_2_, pH 2.5, and nalidixic acid (an antibiotic inhibiting DNA gyrase) supplementation (Wang et al., 2010). The deletion of the entire CPS-53 sequence increases bacterial sensitivity to H_2_O_2_ and pH 2.5, and the individual deletions of *yfdQ* and *yfdS* increase H_2_O_2_ sensitivity (Wang et al., 2010). Similarly, *yfdQ* deletion increases sensitivity to MMS (Rooney et al., 2009). Even though *yfdQRST* deletion did not affect the replication initiation under normal growth conditions in this study (**Figures 3E,F**), YfdQRST- dependent inhibition of replication initiation may assist cell growth under specific environmental conditions, contributing to the maintenance of these genes as part of the cryptic prophage through the evolutionary history of *E. coli*. The localization of CPS-53 in the replication terminus of the chromosome might have biological significance in repressing the copy number of *yfdQRST* genes and thereby reduce YfdQRST expression in normally growing cells. The copy number of the replication terminus in growing cells is lower than that of the replication origin.

Recently, Dps, a protein induced by oxidative stress, was reported to bind to DnaA domain I (Chodavarapu et al., 2008). Also, Dps inhibits *oriC* unwinding *in vitro* and an overproduction of Dps *in vivo* inhibited replication initiation, although the underlying mechanism of these inhibitions remains unknown. Nevertheless, the role for Dps is consistent with the YfdQRST function proposed above. In addition, in *Bacillus subtilis*, SirA protein is known to bind to its cognate DnaA in a Phe49-dependent manner, the residue corresponding to *E. coli* DnaA Phe46 (Jameson et al., 2014). SirA interferes with DnaA–*oriC* interaction, although the mechanism has remained uncharacterized (Wagner et al., 2009; Rahn-Lee et al., 2011). Expression of SirA is induced at the beginning of sporulation, preventing replication initiation. Therefore, a regulatory system might exist with multiple factors using the DnaA domain I as a common target for regulating the replication under various environmental conditions.

## Author Contributions

YN and TK conceived the experiments, YN performed the experiments, and YN and TK analyzed the data and wrote the paper.

## Conflict of Interest Statement

The authors declare that the research was conducted in the absence of any commercial or financial relationships that could be construed as a potential conflict of interest.
