# Supplementary material for: The Escherichia coli Cryptic Prophage Protein YfdR Binds to DnaA and Initiation of Chromosomal Replication Is Inhibited by Overexpression of the Gene Cluster yfdQ-yfdR-yfdS-yfdT
Source: Front Microbiol. 2016 Mar 3;7:239. doi: 10.3389/fmicb.2016.00239 (PMC4776307; doi:10.3389/fmicb.2016.00239)
Supplement: Supplementary file 1 [file Image_1.PDF]

Supplementary Material

The *Escherichia coli* cryptic prophage protein YfdR binds to DnaA and initiation of chromosomal replication is inhibited by overexpression of the gene cluster *yfdQ-yfdR-yfdS-yfdT*

Yasunori Noguchi and Tsutomu Katayama*

***Correspondence:** Tsutomu Katayama: katayama@phar.kyushu-u.ac.jp.

1 Supplementary Figure

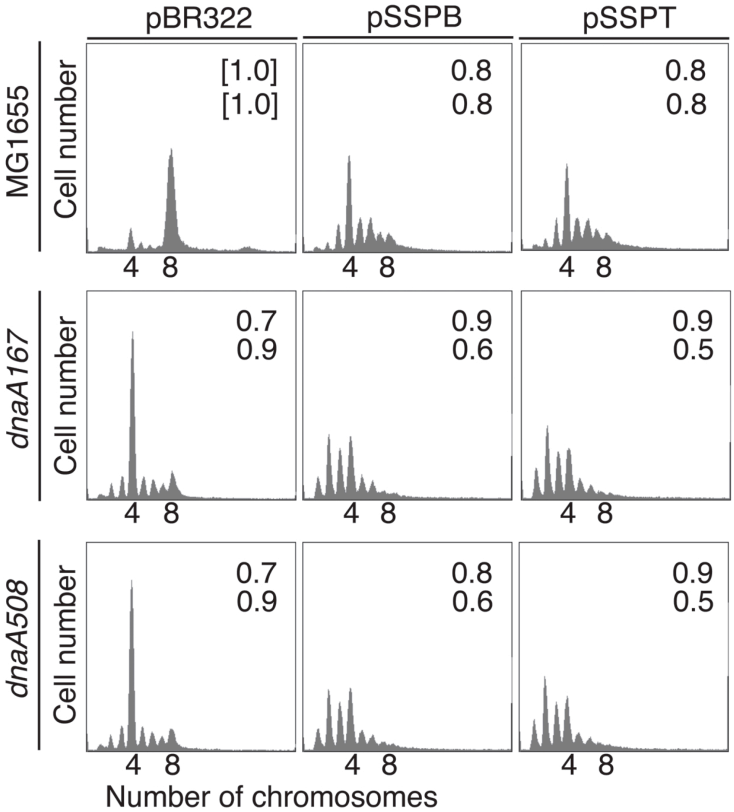

Supplementary Figure 1. MG1655, NY11 (*dnaA167*), or MIT140 (*dnaA508*) strains bearing the indicated plasmids were grown at 30 °C in LB medium, and analyzed using flow cytometry, as described in Figure 2B. Mean cell masses (*upper*) and *oriC* number per cell mass (*lower*) relative to MG1655 cells bearing pBR322 are indicated at the top right corners of each panel.
